# Preventing Post-ERCP Pancreatitis: A Pragmatic Clinical Pathway from Periprocedural Prophylaxis to Early Postprocedural Triage

**DOI:** 10.3390/jcm15103917

**Published:** 2026-05-19

**Authors:** Se Woo Park, Amine Achemlal, Kyong Joo Lee, Dong Hee Koh, Jin Lee

**Affiliations:** 1 Division of Gastroenterology, Department of Internal Medicine, Hallym University Dongtan Sacred Heart Hospital, Hallym University College of Medicine, Hwaseong 18450, Republic of Korea; britnepak@hallym.or.kr (S.W.P.); dhkoh@hallym.or.kr (D.H.K.); jinlee@hallym.or.kr (J.L.); 2 Gastroenterology Unit I, Mohamed V Military Training Hospital, Mohamed V University, Rabat 10045, Morocco

**Keywords:** endoscopic retrograde cholangiopancreatography, post-ERCP pancreatitis, prophylaxis, early diagnosis, risk stratification

## Abstract

Post-endoscopic retrograde cholangiopancreatography pancreatitis (PEP) remains the most frequent and clinically relevant adverse event after ERCP. Although several preventive measures are supported by current evidence, their application in routine practice is often fragmented across the pre-procedural, intra-procedural, and post-procedural phases of care. As a result, patients with evolving risk may not receive timely escalation of prophylaxis or appropriately tailored post-procedural monitoring. This review provides a pragmatic clinical framework for integrating evidence-based PEP prevention with early post-ERCP risk stratification. We summarize baseline risk assessment before ERCP, distinguish routinely applicable preventive measures from strategies reserved for selected high-risk situations, and emphasize the importance of intra-procedural reassessment when procedural events such as difficult cannulation or unintended pancreatic duct manipulation increase risk in real time. We further discuss the role of early symptom assessment and post-procedural amylase/lipase measurement in supporting triage decisions, including selective observation, admission, or same-day discharge in appropriately selected patients. This integrated approach may improve consistency in routine ERCP care while highlighting important limitations related to generalizability, local resources, and implementation.

## 1. Introduction

Endoscopic retrograde cholangiopancreatography (ERCP) is essential in pancreatobiliary management, but post-ERCP pancreatitis (PEP) remains its most common adverse event following ERCP and continues to be associated with substantial morbidity, prolonged hospitalization, and increased healthcare utilization [1,2,3,4]. Over the past two decades, multiple preventive strategies have been supported by randomized trials, meta-analyses, and society guidelines, including rectal nonsteroidal anti-inflammatory drugs (NSAIDs), periprocedural hydration, technical measures to reduce pancreatic duct injury, and prophylactic pancreatic duct stenting in selected high-risk settings [1,5,6,7]. Despite these advances, PEP has not been eliminated, and its prevention remains a major challenge in routine clinical practice.

One reason is that PEP prevention is often approached as a series of isolated interventions rather than as a continuous peri-procedural process. In daily practice, risk is not determined solely before ERCP. Instead, it evolves according to baseline patient characteristics, the planned indication and complexity of the procedure, and intra-procedural events such as difficult cannulation, repeated pancreatic duct access, or the need for advanced access techniques. Post-procedural symptoms and early pancreatic enzyme measurements may further refine the need for observation, admission, or discharge. A static, single-time-point approach therefore does not fully reflect the way PEP risk develops in real-world ERCP care.

Current guidelines provide strong evidence-based recommendations for individual preventive measures but are less explicit about how these measures should be integrated into a pragmatic workflow spanning the pre-, intra-, and early post-procedural phases. The gap in many centers often lies less in the absence of knowledge than in inconsistent implementation, delayed reassessment, and limited integration between prophylaxis and early triage. This is particularly relevant as ERCP practice increasingly emphasizes both patient safety and efficient resource utilization, including selective same-day discharge when clinically appropriate.

**Figure 1:**
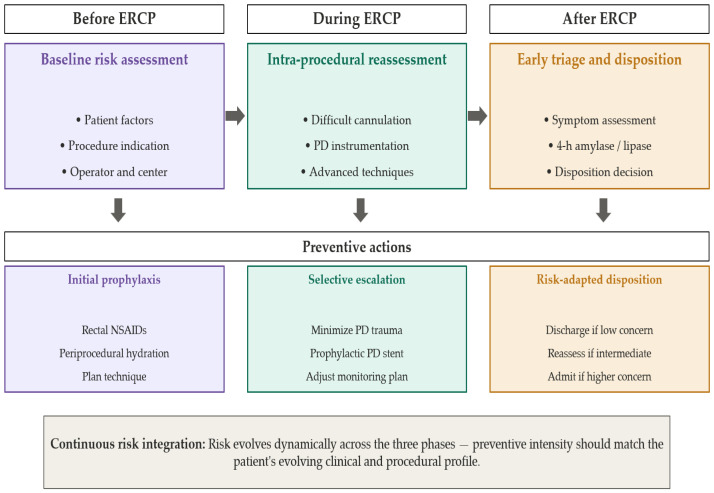
Integrated peri-procedural clinical pathway for PEP prevention and early triage.

In this narrative review, we aim to synthesize existing evidence into a pragmatic clinical pathway for routine ERCP practice. Specifically, we review baseline risk assessment before ERCP, summarize core preventive measures according to routine use versus selective escalation, discuss intra-procedural risk modifiers that should trigger reassessment, and examine the role of early post-ERCP symptom and enzyme-based triage. By linking prevention to reassessment and disposition, we seek to provide an implementation-oriented framework that may support more consistent and clinically actionable PEP prevention; the overall peri-procedural structure of this framework is summarized in Figure 1.

## 2. What This Review Adds Beyond Existing Guidelines

Current society guidelines provide a robust evidence-based foundation for the prevention of PEP and have substantially improved standardization of care. Across major recommendations, there is broad agreement regarding the central role of rectal NSAIDs, the value of appropriate hydration, the importance of minimizing pancreatic duct trauma, and the selective use of prophylactic pancreatic duct stenting in high-risk situations [1,8,9,10]. These documents are highly effective in defining what should be considered evidence-based prevention. However, they are primarily recommendation-oriented and are less focused on how preventive strategies should be linked across the full clinical course of ERCP in routine practice.

In daily care, the challenge is less the absence of knowledge than uncertainty about how to apply preventive measures consistently as risk evolves through baseline characteristics, procedural complexity, and intra-procedural events [2,8,11]. In addition, post-procedural symptoms and early enzyme measurements may further refine the probability of clinically relevant PEP and influence decisions regarding observation, admission, or discharge [12,13,14].

The main contribution of this review is to reorganize existing evidence into a pragmatic, implementation-oriented clinical pathway that links prevention across three phases: before, during, and after ERCP. This framework emphasizes that baseline risk assessment should guide initial prophylaxis but not serve as a fixed estimate; that intra-procedural events should prompt active reassessment and escalation; and that early post-procedural triage should integrate symptoms, procedural context, and biochemical data.

In preparing this manuscript, the authors used an AI-based language model (Claude, Anthropic, San Francisco, CA, USA, version 4.6 Sonnet, 2025) to assist with English language editing, manuscript condensation, and reference formatting. All scientific content, clinical interpretations, and intellectual contributions are solely those of the authors, who have reviewed and take full responsibility for the final text.

## 3. Baseline Risk Assessment Before ERCP

Baseline risk assessment remains the first step in the prevention of PEP. Before the procedure, clinicians should consider patient-related, procedure-related, and operator- or center-related factors that may influence both the probability of PEP and the threshold for applying prophylactic measures [15,16,17,18]. This early assessment is important not only for procedural planning and informed consent, but also for determining whether additional expertise, technical precautions, or post-procedural monitoring may be warranted. In routine practice, pre-procedural risk stratification is most useful when it establishes a structured starting point for subsequent decision-making rather than a fixed label that remains unchanged throughout the ERCP pathway. It should also be noted that the framework presented here primarily applies to ERCP through a native papilla; patients with prior biliary sphincterotomy generally carry substantially lower PEP risk and may warrant separate consideration.

### 3.1. Patient-Related Risk Factors

Patient-related risk factors have been consistently reported, although their relative contribution may vary across studies and practice settings. Younger age, female sex, suspected sphincter of Oddi dysfunction, a history of prior PEP, recurrent pancreatitis, and the absence of chronic pancreatitis are among the features commonly associated with increased susceptibility [15,18,19,20]. These features should lower the threshold for prophylaxis and alert clinicians that even technically uncomplicated ERCP may carry substantial risk. Patient-related factors should not be interpreted in isolation, as their relevance depends on procedural complexity and intra-procedural events.

### 3.2. Procedure-Related Risk Factors

Procedure-related factors are equally relevant at the planning stage. Interventions associated with difficult biliary access, pancreatic duct instrumentation, precut sphincterotomy, balloon dilation of an intact sphincter, or other technically demanding maneuvers may carry greater intrinsic risk, particularly when combined with vulnerable patient characteristics [21,22,23]. Anticipating procedural complexity before ERCP may help identify cases in which preventive planning should be more deliberate from the outset. This may include confirming the availability of rectal NSAIDs, preparing for selective pancreatic duct stenting if indicated, ensuring an appropriate hydration strategy, or considering whether referral to a higher-volume center is warranted in selected situations [5].

### 3.3. Operator- and Center-Related Factors

Operator- and center-related factors should also be recognized as part of baseline risk assessment, even though they are sometimes underrepresented in conventional risk models. Procedural volume, familiarity with advanced cannulation techniques, experience with prophylactic pancreatic duct stenting, and institutional workflows for post-ERCP monitoring may all influence the reliability with which preventive strategies are implemented [24,25,26,27]. A prophylactic approach that is feasible in a high-volume tertiary center may not be directly transferable to a lower-resource setting without adaptation. For this reason, baseline risk assessment should include not only the characteristics of the patient and the planned intervention, but also the practical context in which the procedure is being performed.

Table 1 summarizes the major baseline risk domains and their practical implications for routine ERCP care. The purpose of this table is not to create a rigid scoring system, but to provide a clinically usable framework that links known risk factors to preventive planning and procedural vigilance.

Importantly, pre-procedural risk stratification should be viewed as a practical starting framework rather than a definitive prediction model. A patient initially classified as lower risk may become high risk after repeated pancreatic access or difficult cannulation, whereas an apparently high-risk case may proceed uneventfully. The purpose of baseline assessment is therefore to establish an initial preventive strategy and identify cases requiring heightened procedural awareness (Figure 1).

## 4. Core Preventive Measures: Routine Measures and Selective Escalation

Preventive strategies for PEP are most useful when organized according to whether they should be applied routinely or reserved for high-risk situations. The goal is to ensure consistent use of broadly supported measures while preserving a lower threshold for escalation when risk increases, thereby avoiding both underuse of effective prophylaxis and indiscriminate use of technically demanding measures.

### 4.1. Rectal NSAIDs

Rectal NSAIDs remain the most consistently supported pharmacologic strategy for PEP prevention and should be considered a foundational component of routine prophylaxis in the absence of contraindications [5,28,29,30,31,32]. Their role is particularly important because they are simple to administer, relatively inexpensive, and applicable before procedural complexity fully declares itself. In this sense, rectal NSAIDs are well suited to serve as a baseline preventive measure across a broad spectrum of ERCP indications rather than as a rescue intervention applied only after procedural difficulty has already emerged.

### 4.2. Periprocedural Hydration

Periprocedural hydration is also a core preventive measure, although its implementation is inherently more context-dependent than that of rectal NSAIDs [6,32,33,34,35]. Adequate hydration may support pancreatic microcirculation and attenuate inflammatory injury, but its practical value depends on patient selection, fluid type, timing, and tolerance [6,36,37,38]. For this reason, hydration should be approached as a structured component of prophylaxis rather than as routine volume expansion applied uniformly to all patients. Individuals with cardiac, renal, or other conditions limiting fluid administration require individualized planning, and the intended preventive benefit must be balanced against the risk of fluid overload. When hydration is employed, lactated Ringer’s solution is generally preferred over normal saline based on mechanistic rationale and supportive data from acute pancreatitis literature [39]. Standard hydration (approximately 1.5 mL/kg/h during and for 8 h after ERCP) is commonly used, while aggressive protocols have been investigated in selected high-risk situations [6,40,41]. Notably, the FLUYT trial—a multicenter randomized controlled trial—demonstrated that aggressive hydration did not provide additional benefit in patients already receiving rectal NSAIDs [42]. Accordingly, aggressive hydration should not be applied uniformly, and its use should be balanced against the burden of fluid administration and the risk of volume overload, particularly in patients with cardiac, renal, or advanced-age comorbidities.

### 4.3. Procedural Technique

Routine prevention also depends on procedural technique. Although technical measures are often discussed separately from pharmacologic prophylaxis, careful cannulation strategy, avoidance of unnecessary pancreatic duct trauma, and early recognition of difficult access are integral to PEP prevention [1,11]. These are not secondary considerations but central determinants of whether the theoretical benefit of other preventive measures can be fully realized. Accordingly, routine prevention should be understood as a combination of baseline pharmacologic prophylaxis and technically disciplined ERCP performance from the outset of the procedure.

### 4.4. Selective Escalation: Prophylactic Pancreatic Duct Stenting and Combined Prophylaxis

In contrast to routine measures, some preventive strategies are most appropriately reserved for patients whose risk is elevated by baseline features, procedural intent, or intra-procedural events. Prophylactic pancreatic duct stenting is the clearest example. While pancreatic duct stenting is highly relevant in selected high-risk situations, it is not a universal measure and should not be presented as such [43,44]. Its benefit is closely linked to technical feasibility, endoscopist expertise, and the clinical setting in which pancreatic duct access has already occurred or is anticipated. The decision to place a prophylactic pancreatic stent should therefore be made within a selective escalation framework rather than as a blanket extension of routine prophylaxis [10,12].

Selective escalation may also include intensification of hydration, a lower threshold for extended post-procedural monitoring, or early planning for admission when baseline risk is high and the procedural course becomes concerning [1,10,45]. Importantly, escalation does not necessarily mean adding every available intervention. Rather, it means matching the intensity of prophylaxis to the evolving risk profile of the individual case [46]. A patient with multiple susceptibility factors and repeated pancreatic duct manipulation may reasonably require a different preventive strategy from a patient with uncomplicated biliary cannulation, even if both were initially scheduled for ERCP under similar indications.

Table 2 provides a practical overview of the core preventive measures discussed in this review, distinguishing interventions that should generally be considered part of routine prophylaxis from those most appropriately used in selected high-risk situations.

### 4.5. Other Proposed Strategies

Other proposed preventive strategies may be relevant in selected contexts but should not be assigned the same weight as interventions supported by stronger evidence. For example, sublingual nitrates and somatostatin analogs have shown inconsistent or marginal benefit in meta-analyses [47,48], while allopurinol, N-acetylcysteine, and protease inhibitors have not demonstrated robust efficacy in adequately powered trials [49]. The limited role of these agents reflects the difficulty of translating mechanistic rationale into clinically meaningful risk reduction in the heterogeneous real-world ERCP population.

This distinction also clarifies a practical principle: rectal NSAIDs and hydration are generally most useful when implemented early, while pancreatic duct stenting and other escalation measures depend more directly on procedural events [11].

## 5. Intra-Procedural Reassessment and Escalation of Prophylaxis

A central limitation of static risk models is that they do not fully account for what happens during ERCP itself. In many cases, the most important determinants of PEP risk emerge only after the procedure has begun. For this reason, effective prevention requires active intra-procedural reassessment rather than reliance on a pre-procedural estimate alone. Procedural events such as difficult cannulation, repeated pancreatic duct access, guidewire passage into the pancreatic duct, contrast injection, or the need for advanced access techniques should not simply be documented retrospectively; they should function as real-time triggers for reconsidering the preventive strategy [1,8,50].

### 5.1. Difficult Cannulation

Difficult cannulation is among the most clinically relevant intra-procedural warning signs because it is closely linked to repeated instrumentation, prolonged manipulation at the papilla, and a higher likelihood of pancreatic duct trauma [50,51,52,53]. Even when biliary access is eventually achieved, the route to that endpoint may substantially alter the patient’s risk profile. Accordingly, procedural success should not be interpreted as evidence that risk has remained low [22,54]. The manner in which access is obtained is often as important as whether access is ultimately achieved.

### 5.2. Pancreatic Duct Instrumentation and Opacification

Repeated guidewire entry into the pancreatic duct or unintended pancreatic duct opacification should similarly prompt reassessment. These events are not merely technical inconveniences; they may indicate a procedural course in which pancreatic irritation has already occurred and in which the threshold for escalation should be lower [46,55,56]. In such settings, endoscopists should consider whether prophylaxis initially planned on the basis of baseline risk remains sufficient or whether additional measures, including selective pancreatic duct stenting or intensified post-procedural observation, have become more appropriate.

### 5.3. Advanced Cannulation Techniques

The need for precut access or other advanced cannulation techniques frequently marks a transition from anticipated risk to realized procedural risk [57,58]. These techniques may be necessary and effective, particularly in experienced hands, but they should be recognized as part of the dynamic risk environment of ERCP rather than as isolated technical decisions. When advanced access becomes necessary, prophylaxis should be reconsidered in parallel. In a pragmatic pathway, the procedural strategy and the preventive strategy should evolve together rather than on separate tracks.

This point has direct implications for post-procedural planning. Escalation during ERCP may involve not only selective pancreatic duct stenting, but also a conscious shift in the post-procedural plan. A patient whose baseline profile appeared to be moderate risk may warrant prolonged observation, early enzyme testing, or a lower threshold for admission after a challenging procedure [15,59]. Conversely, the absence of major intra-procedural risk modifiers may support a less intensive post-procedural pathway when other clinical parameters are reassuring. In this way, intra-procedural reassessment serves as the bridge between baseline planning and post-procedural triage.

### 5.4. Integrating Intra-Procedural Findings into Post-Procedural Planning

A practical prevention framework should therefore treat real-time decision-making as a core component of prophylaxis rather than as a separate procedural consideration. This requires more than technical awareness; it requires a systematic approach that recognizes when risk has changed and triggers timely modification of the original plan.

Importantly, escalation should remain selective and clinically reasoned. The purpose of intra-procedural reassessment is not to trigger reflexive overuse of every available preventive measure, but to support proportionate responses to events that meaningfully alter risk [8,60]. This balance is essential in routine practice, where technical expertise, equipment availability, patient comorbidity, and local workflows all influence what constitutes appropriate escalation. A well-functioning preventive strategy is therefore not only evidence-based, but also adaptive.

**Figure 2:**
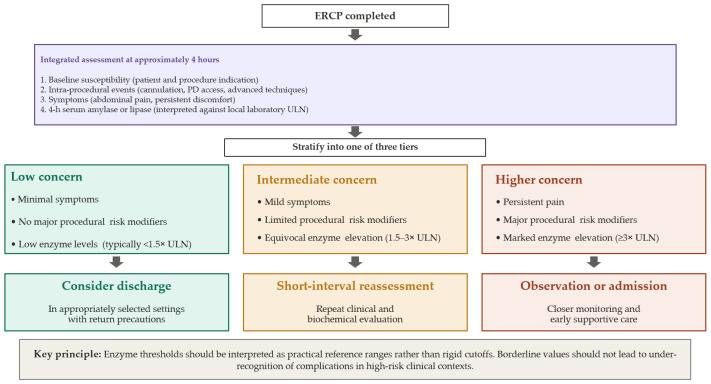
Early post-ERCP triage and disposition algorithm (~4 h).

This evolving risk profile is most clinically useful when it informs the early post-procedural phase, in which symptoms, procedural events, and biochemical markers can be integrated to guide observation, admission, or discharge. Because this evolving procedural risk directly informs early disposition decisions, it also provides the clinical bridge to the vertical triage algorithm shown in Figure 2.

## 6. Early Post-ERCP Triage and Disposition

Early post-procedural evaluation represents the final step in a practical peri-procedural prevention pathway. Even when preventive measures have been appropriately applied, the immediate post-ERCP period remains clinically important because it is the stage at which evolving symptoms, procedural events, and biochemical markers can be integrated to identify patients who require closer monitoring and those who may be considered for discharge in appropriately selected settings [2,15,61]. This final step of the pathway is presented in detail in Figure 2, which organizes early post-ERCP triage as a vertical algorithm integrating baseline susceptibility, procedural events, symptoms, and early enzyme measurements.

### 6.1. Rationale for Early Triage

Traditional delayed assessment pathways may be unnecessarily restrictive for some patients while still insufficiently individualized for others. In contrast, an early structured assessment at approximately 4 h may help refine post-procedural concern in a more clinically actionable way [13,62]. In the revised algorithm, early symptoms, procedural modifiers, and 4 h enzyme levels are interpreted sequentially within a single integrated assessment step, emphasizing that biomarkers should support rather than replace clinical judgment. This approach is particularly relevant because the significance of an elevated amylase or lipase level depends on the broader peri-procedural context, including baseline susceptibility and the degree of intra-procedural pancreatic manipulation. This timing is supported by consistent diagnostic performance data. A meta-analysis of 18 studies (11,790 ERCPs) demonstrated that serum lipase measured at 2–4 h post-ERCP yielded the highest pooled sensitivity (92%) for PEP, while amylase in the same window provided the highest specificity (93%) [13]. Earlier prospective work by Lee et al. (*n* = 516) similarly reported areas under the curve of 0.919 and 0.933 for 4 h amylase and lipase, respectively [14], and a recent multicenter prospective study confirmed these findings with AUCs of 0.877 and 0.893 [15]. The 4 h window therefore reflects an evidence-based balance between diagnostic accuracy and clinical actionability rather than an expert-derived cutoff.

### 6.2. Biochemical Assessment

Although specific cutoff values vary across studies and assays, serum amylase or lipase levels exceeding 3 times the upper limit of normal at 4 h post-procedure have been commonly used as a threshold for heightened clinical concern. From this integrated assessment, patients can be grouped into low-concern, intermediate-concern, or higher-concern pathways, which in turn support consideration of discharge, short-interval reassessment, or observation/admission, respectively (Figure 2). Patients with minimal symptoms, no major procedural risk modifiers, and low early enzyme levels may be considered for discharge in appropriately selected settings. By contrast, patients with mild symptoms, limited procedural modifiers, or equivocal enzyme elevation may benefit from short-interval reassessment, including repeat clinical evaluation and selective repeat testing when needed. Patients with persistent pain, major procedural risk modifiers, or marked enzyme elevation should undergo closer monitoring and early supportive management, with admission as clinically indicated. These three tiers—low concern (typically <1.5× ULN), intermediate concern (1.5–3× ULN), and higher concern (≥3× ULN)—should be interpreted as practical reference ranges rather than rigid cutoffs, given the variability across studies and assays [15,63,64]. Importantly, the 3× ULN threshold should be applied based on the reference values provided by each local laboratory, as normal ranges vary between assays and institutions. This explains in part why reported cutoffs may appear different across studies. Equally important, biological markers should not be interpreted in isolation but as part of a comprehensive clinical assessment. Slight variations below the threshold (for example, 2.7–2.9× ULN) should not lead to under-recognition of an early complication, particularly in high-risk patients, after high-risk procedures, or when the post-procedural clinical examination is concerning. The 3× ULN threshold is itself data-supported: in the Hirota et al. meta-analysis, this cutoff yielded pooled sensitivities of 71.1% (amylase) and 85.8% (lipase), with specificities of 91.2% and 85.3%, respectively [63]. However, the operational integration of this threshold into a three-tier framework, including the 1.5–3× ULN intermediate-concern zone and guidance for borderline values, represents an evidence-informed expert framework rather than strictly validated cutoffs.

### 6.3. Safety-Netting and Discharge Planning

A pragmatic early triage strategy should also incorporate safety-netting. Even patients considered for discharge should receive clear return precautions, written discharge instructions, and guidance regarding warning symptoms that should prompt urgent reassessment. Accordingly, Figure 2 should be read not as a rigid discharge rule, but as a structured early post-ERCP disposition algorithm in which symptoms, procedural context, and biomarker findings are interpreted together. Used in this way, early triage may improve alignment between peri-procedural risk and the intensity of post-procedural care while preserving the central role of clinical judgment.

Table 3 summarizes a practical framework for early post-ERCP risk stratification and disposition, integrating baseline risk, intra-procedural events, symptoms, and early biochemical findings.

This structured post-procedural approach reinforces the broader principle that PEP prevention is most effective when treated as a peri-procedural continuum rather than a series of isolated steps.

The overall structure of this integrated peri-procedural framework is summarized in Figure 1, which condenses the clinical logic of PEP prevention into three linked moments: baseline risk assessment and prophylaxis planning before ERCP, active reassessment and selective escalation during ERCP, and early post-procedural triage based on symptoms, procedural events, and 4 h enzyme levels. The detailed logic of the final triage step is expanded in Figure 2, which provides a more operational disposition algorithm. Together, Figure 1 and Figure 2 serve complementary roles: Figure 1 provides the peri-procedural overview, whereas Figure 2 translates the post-procedural component into structured clinical decision-making.

## 7. Practical Limitations and Unresolved Questions

Although a pragmatic peri-procedural pathway may improve the clinical organization of PEP prevention, several limitations should be recognized. First, not all components of the pathway are supported by the same level or type of evidence. Some measures, such as rectal NSAIDs, are broadly supported and readily incorporated into routine care, whereas others depend more heavily on procedural context, technical expertise, or local feasibility. A pathway-based framework should therefore not be interpreted as implying equivalent certainty across all steps. In particular, the applicability of this framework may differ between patients undergoing ERCP for malignant biliary obstruction and those with benign disease, and between native-papilla and post-sphincterotomy procedures. The principles of dynamic risk integration apply broadly, but specific risk thresholds and disposition decisions should be calibrated to the clinical indication and patient context.

Second, the generalizability of early post-ERCP triage strategies remains an important consideration. The interpretation of early amylase or lipase levels may vary according to assay thresholds, patient selection, procedural complexity, and institutional practice patterns. Biomarker-guided triage is likely to be most useful when applied within a broader clinical context rather than as a rigid standalone rule. External validation across diverse practice environments remains important, particularly if early discharge pathways are to be used more widely.

Third, implementation itself is a major unresolved issue. Even when evidence-based preventive measures are known, adherence may remain inconsistent because of differences in operator preference, nursing workflows, medication availability, post-procedural logistics, or local thresholds for observation and admission. The effectiveness of any prevention pathway therefore depends not only on the scientific merit of its individual components, but also on the extent to which it can be incorporated into real clinical systems. In this regard, quality-improvement initiatives, checklists, and standardized peri-procedural workflows may be as important as the interventions themselves.

In centers with limited resources and without access to rapid laboratory results, a careful clinical assessment becomes even more important. In such situations, we recommend a thorough patient interview and close physical examination, especially in patients at higher risk (as detailed in Figure 2), to ensure timely recognition and appropriate management of these complications.

Finally, important questions remain regarding how best to individualize prevention. Not all patients with risk factors develop PEP, and not all patients who develop PEP are clearly identifiable before ERCP. Future research should therefore focus not only on refining predictive performance, but also on improving clinical usability. This includes determining which combinations of baseline factors, procedural events, symptoms, and biomarkers are most informative; how such information can be operationalized in different care settings; and how pathway-based prevention can be implemented without encouraging unnecessary intervention or prolonged observation.

## 8. Conclusions

PEP prevention should not be viewed as a single intervention applied at a single time point. Rather, it is best approached as a peri-procedural clinical pathway that begins with baseline risk assessment, requires real-time intra-procedural reassessment, and extends into early post-procedural triage. Rectal NSAIDs, appropriate hydration, careful technique, and selective prophylactic pancreatic duct stenting remain central elements of evidence-based prevention, but their value in practice depends on whether they are applied consistently and escalated appropriately when procedural risk changes.

Early post-ERCP evaluation, including symptom assessment and timely enzyme measurement, may further improve decision-making by identifying patients requiring closer monitoring and those suitable for discharge. However, this approach should be interpreted within the broader clinical context, recognizing variability in local practice, biomarker thresholds, operator expertise, and resource availability.

In summary, this review integrates current evidence into a pragmatic, implementation-oriented framework for routine ERCP care. Future work should focus on external validation of early triage strategies, adaptation across diverse practice environments, and quality-improvement efforts that translate evidence-based prevention into more reliable bedside practice.

## Figures and Tables

**Table 1:** Key Risk Factors for PEP and Their Practical Implications.

Domain	Key Factors	Practical Implication
Patient-related	Young age, female sex, suspected sphincter of Oddi dysfunction, prior PEP, recurrent pancreatitis, no chronic pancreatitis	Lower threshold for routine prophylaxis; anticipate that apparently straightforward ERCP may still carry substantial risk
Procedure-related	Difficult biliary access anticipated, pancreatic duct instrumentation, pancreatic contrast injection, precut techniques, balloon dilation of intact sphincter	Pre-plan preventive strategy; consider selective escalation if technical difficulty is expected or likely
Operator-/center-related	Lower procedural volume, limited experience with advanced access or PD stenting, variable post-ERCP monitoring pathways	Adjust procedural planning to local expertise and resources; consider referral or adaptation of pathway where appropriate

PEP, post-ERCP pancreatitis; ERCP, endoscopic retrograde cholangiopancreatography; PD, pancreatic duct.

**Table 2:** Evidence-based preventive measures for post-ERCP pancreatitis.

Preventive Measure	Usual Role in Practice	Practical Caveat
Rectal NSAIDs	Routine baseline prophylaxis in eligible patients	Avoid or modify use when contraindications are present
Periprocedural hydration	Routine structured support, tailored to patient tolerance	Requires individualized planning in patients with cardiac/renal limitation
Careful cannulation technique/minimization of pancreatic injury	Routine technical prevention	Depends on operator judgment and early recognition of procedural difficulty
Prophylactic pancreatic duct stenting	Selective escalation in high-risk settings	Requires technical expertise, appropriate indication, and feasibility
Other or emerging measures	Limited or evolving role	Should not displace established interventions with stronger evidence

NSAIDs, non-steroidal anti-inflammatory drugs.

**Table 3:** Practical framework for early risk stratification and triage after ERCP.

Clinical Situation	Typical Features	Suggested Disposition
Low-risk early recovery	Reassuring symptoms, uncomplicated procedural course, low early enzyme levels (typically <1.5× ULN)	Consider discharge with clear return precautions in appropriate settings
Intermediate concern	Mild symptoms and/or limited procedural risk modifiers, equivocal enzyme elevation (typically 1.5–3× ULN)	Observe longer and reassess clinically and biochemically as needed
High post-procedural concern	Persistent pain, difficult cannulation, repeated pancreatic duct access, marked enzyme elevation, other concerning clinical features (typically ≥3× ULN)	Admit or intensify monitoring/supportive care according to clinical context

## Data Availability

No new data were created or analyzed in this study. Data sharing is not applicable to this article.

## Abbreviations

The following abbreviations are used in this manuscript:

ERCP	Endoscopic retrograde cholangiopancreatography
ESGE	European Society of Gastrointestinal Endoscopy
PEP	Post-ERCP pancreatitis
NSAIDs	Nonsteroidal anti-inflammatory drugs
PD	Pancreatic duct
SOD	Sphincter of Oddi dysfunction
ULN	Upper limit of normal
